# Supplementary material for: Association Analysis of Tiller-Related Traits with EST-SSR Markers in Psathyrostachys juncea
Source: Genes (Basel). 2023 Oct 21;14(10):1970. doi: 10.3390/genes14101970 (PMC10606050; doi:10.3390/genes14101970)
Supplement: Supplementary file 1 [file genes-14-01970-s001.zip › Table S1.pdf]

Table S1 SSR primer information

Primer	Upstream primer	Downstream primer	Annealing temperature (℃)
000081	TCACTGCCTCTGCATCAATC	CCTCGGAGAAGCATGTGAAT	55.0
000516	CCAACGTCCATCCTTGAAGT	ACGGTTTGAACTGTTTTGCC	54.5
002249	GCGCACAGAGTTAATGGTGA	GTGAAGGGAAGAAGTGCCC	56.0
002947A	ATCCTCCAACTTCCCAGTCC	AAGCAAGGGGAGAAGAGACC	57.0
004435	ATCTCGCCTCCTCTCTCCTC	GTTTCCACCTCCTCCTCCAC	58.3
006022	TTCCCAAACGGATCAAGTTC	AAAATTTGAGGCAAGCGTGA	52.5
006181A	CCTTTTCCGTGCATACTGGT	CTGCGAGGGAATGATGGTAT	55.0
008649	GGCACTTATGACACCCCAAG	GGCGTGTGTATGCGAGTATG	56.4
013109	TCCTTCGGGATCCCTCTACT	ATGGTAGGGTTGGCTCTGTG	57.0
013558	ACTAGCGAAGCCCTGTCAAA	TCCCTAACTTCCTTCCCGTT	56.0
016533C	TAGGTCAAGCCCAAGTTGCT	TCTTTACCGTCACCTCAGCC	57.0
018679	GCGACGGGAAGAAGAACTC	GGGTAGCCATGAGCACAAAG	56.5
019368C	TGGATTGTTCTAGCTGCGTG	ACCTGTGTCTGTCCATTCCA	55.5
022073A	CTGTCCCATCTCCCCTGTAA	CACTAGGAGGTCGGGCATAA	56.4
022271	GGTTCTTCCTTCCTTCCTCG	AGGGTAAGGGCCAGGTTTTA	55.5
024537A	AACAGCCTCTGTTTGCTCGT	CCACCTCGTCCAGTCATTCT	57.0
025029	ACTGATATACCCGGTCGCTG	GGCCGGAGATTAGGCTTTAC	56.5
026249	CTGACCTGCATTCAGAGCCT	AGTACAGCATCCCGACCAAC	57.5
026620	CAACCTGTTGGGTTGGTAGG	CTCCCCAAGAAGCACGTATG	56.0
027806	GCAGTGTACATTTGCGCAGT	AACACTGACATGTGCAAGCC	56.3
028161A	GCCCTACCTTAGCCCCTTT	GGCTCACTCACGATGGAGAT	57.0
028324	ATGAAGAGAATTTGCGGGTG	AATTTTGTAGGCGAGACGGA	53.2
028410	AAATGGAGAGGGGTTGTGTG	CACGCAGACATTGGAGTCAT	55.0
028553	TTGACAAATCAGTCAGGCCA	ACACCGAGAAATCCCATCAC	54.5
029223A	GTCGTGATCCCTGGTCATGT	ACGACTTCTTCTCGCAGCAT	57.0
029223B	TGGTACATGTGTCCACCACC	AAGAGCATCCTCGACAGCAC	57.5
029697	AGGCACAACTTGCTGCATAA	GCCACAACCCCATTAACAAC	54.7
030766B	CTCTTGTGGCCCGGTATAAA	GGCATACCTGCTTGACGAAT	55.0
035661	TACGCACCATCACCACTTGT	CGACGACGACTACGAGTTCA	56.6
037119	CGCTTTATACTCCCGCAGTC	CATCGTAGGTGCACAACACC	56.3
037353	GAAGCTCTGGTTGTTGGAGG	AGCCTTCTTCTTCCTCCTGG	56.5
038976	GTTTAGGCGGGAGAGAAACC	CACCATCTCCCACAGCCTAT	56.5
040957	TGTTACGCCCCCAAAGTTAG	CGCCATAGCTTCAGTCAACA	55.0
043242	CGTGATGAAAACGCGTAAGA	GATGTTCATGGCAGCCTTTT	53.2
043637B	GTGTGGCGATTCATGTTGAC	CAGAACGAAGATGTGCTCCA	54.7
044262	TACGACTTCCTCGAACACCC	GTCCAGTCGTCGATCTCCTC	57.2
045589	GACAGATTTGACTGGGAGCC	AAGGAACTCTTGAGTCCGCA	56.0
045600A	TCTCGTAGACCGCCTTGAAT	GCCACCATAGCTTGCTTCTC	56.0
046829B	GATCATGGGGAAGCGCAC	AACAACGACTCTCACCCACC	57.2
051628	CTTAAGTTGCATGTCCCCGT	TGGTCCATTCTCTTGGGAAG	54.7

Primer	Upstream primer	Downstream primer	Annealing temperature (℃)
058749A	CCTCGTAGAAGACGTAGGCG	GACTTCAAGGCGCTGGTG	57.5
060437	GAAGAACAGGGACTGGACGA	TGGGGAAGAGTCTCACTTGG	56.6
061356	GTGTGCTACGCTTGGGTGT	CGTCCTCACTCTGTGGCTC	58.5
063559	ACATTTCCAGCCACATCCAC	AAGCCTTGTCCGTCTTGTTG	55.6
064748	GCCTGAAATCCAAGCACATT	CCCTGTTTAATCGGCTACCA	54.0
065010	GAACAGACGAAACCGCCC	TGGTGAGACTCTGCTCATGG	57.0
065544	GAGGGCGAGCCAAATAATC	ATGCTTCATTTGTTAGCGGG	53.4
066628	TGTGTTGGCCCGAACTAATA	TTCAATACAAGCCATTCAGGC	53.4
066842	AAGGTAGGGCCATTGTACCC	CTGGGTCATGAACGACTCCT	57.0
067939	GATCGGATTGTTGCTAGCCT	CCTTCCACTTCTTCTCGCAG	55.4
068444	CGAAGTCAAAAGCGAACACA	AATCATGGATCCCACCTGAA	53.1
069862	GGAGGTGGATCTACCGGG	GTGGGAGAAGACGACCATGT	57.5
070047	GTAAGCGGCAACCTCAGAAC	AACGTCTTATCCTCCTGCCC	56.8
070343	TTCTCTCACTGCACCCATTG	GCACCTTGCATTGCTGTCTA	55.4
071974	CTTGGCCTTGACGACCTTAG	GAAGTATGCCGACCTGAAGC	56.0
072525	ATCTTGCTCGAACGAACCAC	TGAAACCCCTTCTTCCTCCT	55.5
072655A	TGTGGTACTACGCCTTGCTG	GACAGTAGAGCCAGCTTCCG	58.0
073069	AAACAAACTGTCCCTCACCG	CTTTCTGGCTCAGCACTTCC	55.8
073311	CACCCACCATAGCGAGTTG	GGGAAGTTGCTCTTCAGCAC	56.5
073848A	TGGATACAATCCGTCTGCAA	GTATCACGGGCCAATTCATC	53.5
074428A	TGGACAGCGATCTTTCACAG	GCCTCGTTGTCCAGATTGTT	55.2
074564	TCGGCTTCCTCTTCCTATCA	CAGGAGCTCCAAACCACTTT	55.0
075415B	CCTCTCCACCCTGTCTGTTC	CTCTCTTCTTCGACCGTCCC	57.7
075587A	GGTCAAGAATGGAGACTCGC	TCCTCTTCATTGAGGTCGCT	55.8
094279	AGACACGAGCAGTGCAGCTA	CTGCCCAGCTGTAAGCAGT	58.5
099009	TTGTCTGTTCTGGGGTCCTC	AGCAGAACCGATGACTTTGC	56.5
114416	GACGAGCCTGATATGTGCAA	CTTTATAGCCGTCCGGTGAC	55.5
120572	ACAAAGACGCCAATCCTGAC	CATCTGGATCCACTCGTCCT	56.0
126480	CACCACAGGAGATGCTGCTA	GGCGAGTCTGTTTTGGAGAC	56.8
127259	TCCTTCGGGATCCTACCTTT	GCTTGTGTTGATGCCTCTGA	55.2
128934	TTCGAAAGAGGAATTTTGCG	ACCAACCAAATCCAAATCCA	51.1
132067	TAAAAGAGGCATTGGTTGCC	ATGTTGCCGACATAGCATCA	53.5
132162	GTTGGCCACATTCCCTAAGA	TCAACGTTGCAGGTTACAGC	55.3
133160	AGTTCTTCAGCTCGACCCAA	GGTCCTTGAGGGTGAAGTTG	56.0
134436	TCTTTCTCTCCACACCCAGC	TGAGCTAGGACTCGCCAGAT	57.5
135127	CCGCAGTTGACATTCTCCTT	TATCCTTGGGGACTGGAACA	55.2
137131	GTTGTCGAGCCGGAACTTCT	TATCCACACTGTCGAGCTGC	57.5
138171	ATTCAACGCTGTAGCCCATC	GGTATTCTGCCTTTGGGTGA	55.0
139768A	TCCGACATGTCATCCTTTGA	TTTCTGTCAACATCTGCATGG	53.1
139898	TCTCTGCTGTCTTCCCCTGT	GTCAAAGCAGAGAGCAGCCT	58.0
139974	GGTAGCAAAGTTTCCCATGC	AACAGCAATGAGCTCCGTTT	54.7
140425	GCATGCTATGGGCACAACTA	CTGGTCTGCACTATGCCAAA	55.3

Primer	Upstream primer	Downstream primer	Annealing temperature (℃)
141012	CTTCTTCTCCTCCTGATGCG	GGAGATCCTTCAAGTGCGAG	55.6
141141	AATTTCTTGGCGTGTTCCAC	CCAAATCGAATTGCTGTCCT	52.8
142295B	GGAACGAGGAGGAGGAAGAC	GTATCTGAAGTCGGCGTCGT	57.4
142346	TTTCAGCTCGACCTCTTCGT	GAGATGGGTGAGGAGGAACA	56.3
142484	CCAACGTGTCTAGCTCCCTC	ACGTCACCCAGCTTGAGACT	58.5
142585	CTCCTGCTTTCTCGCTCG	GAACGTGTCCCAGATGGTCT	56.8
142612A	GTTGCACAAGAACGCAAAGA	TCTTCATCCCCTTTGTCAGG	54.0
143409	GAAGAACCTTGCTGAGCGTC	GCTCGGTTATCTGCCTCTTG	56.3
144127	GAGCATGGTGGTGCAGATAA	GGATGCATGTTAGGGCTGTT	55.3
144827	AGTCTTCTTCCTCCCCTTGC	CATCTTCTGCAGCAGTCCCT	57.4
144926B	TGGTGAGTTTACTCCCCCAC	ACCAGAATGCTGCTGCTCTT	57.0
146991	GGATGTAGGAGACCGCCAT	ACGAAGACGAAGGCTGGAT	56.8
149977	GATGATGACTGGATCACCCC	ACGCAGAGGAGGAAACTCAA	55.3
163142	TAGCAATTTGGGAAGGCATC	TCATTTTTGGAGCAGGGTTC	52.8
163639	GTATGGGGTGGCTTTCCTG	GTTGCTCCAACTCTTGAGCC	56.3
165982	ACGACCAGCACGTTCTTCTT	CCACTCATCACTCCCTCCTC	57.0
167041	GACCCTCAAGTTGCAATGGT	CCGCAAAGATGACACAGAGA	55.2
185875	GCCTGTAGTCTCGTCTTGGC	TACCTCTTCTGCTGCCACCT	58.5
193064B	GCATCGATGACGACAGGAG	GATCTGGTGTCCTTCGGTGA	56.3
194430	GTCGTGGCGTCTACCCATAG	GAACGACGCACACTGGTTC	57.7
194938	GTTCCTCATTTGGGCGAGTA	GCACGGCATCTTTTAGCTTC	54.6
OsIPT4	TGGATGTGGTGACGAACAAGGTGAC	GATCTACGTCGACCCAGAGGAAGCA	62.0
OsIPT5	AGGTGATCAACGCCGACAAGCTGCA	TCGACGAGCTCCTCGATGTAGGAGT	63.5
OsIPT7	TGGACGACATGGTGGACGCTGGCAT	GCTTTGATGTCGTCGATCGCCTCGG	65.0
OsIPT8	GTCGACGACGATGTTCTCGACGAAT	TGTTGGCCTTGATCTCGTCTATCGC	61.0
OsCKX1	ACAAGGCGTACCTGGCGCAC	TGGCCAGGGGAGAGCAGCTT	64.5
OsCKX4	GCCACAGGACCCAGTGCAGG	TTCAGCCACGGGTGTGGGACT	64.5
OsCKX5	CGCTGCTGGGCGAGCTGAAT	CGCCTTGTGCACGCGGTCTA	64.4
OsCKX9	GCCAGGATTCCTCTTGAACCTGC	ACGCACTGGGTCCTGCGGAT	61.0
OsYUCCA4	GCAGAATGGCCTGTACGCTGTTGG	CAGACCAGCACATGACGTGTCTAC	63.5
OsYUCCA6	CCATTCCCAGATGGTTGGAAGG	CATGTTGCGCCTCAAGATATTTG	57.5
OsRR6	CCGAGGACTTCCTGCTCA	TCATCCTCTCCATGATCCAA	53.5
OsRR7	TGCTCAAGAAGATCAAGGAATCG	GGCACGTTCTCTGACGACATTAT	56.0
OsRR11	CTAGGCTCGGAACCAAATGT	ACGGGGATCTTCTTCAGCTT	56.0
OsTB1	GCCGGATGCAAGAAATC	TCAGCAGTAGTGCCGCGAA	55.5
D3	GCAGTTGCGGGAGGACTATT	CACGCCATCCCATTTGTCAC	57.4
D14	TCTCCCCGGTTCTTGAACGA	CGTCGAACACCTGCTGTATCT	58.5
OsD3	TTCGGCCTACTCTCAAGGAA	CACAGCTTCACTGAGGTCCA	56.5
OsD10	GGTAGCAACGAGAGGCAGTT	TCGACCTTGGTGAGCGTGTT	59.0
OsD27	TCTGGGCTAAAGAATGAAAAGGA	AGAGCTTGGGTCACAATCTCG	55.5
OsD17	ACCTCGTCCAGAAGCGTGAGT	AGGCCCAGTCGTGGATCA	59.5
OsMADS57	TATCCATCCTCTGCGATGCG	GAAGTTGCATTCCCGCCAAG	57.4

Primer	Upstream primer	Downstream primer	Annealing temperature （℃）
OsPIN1a	TCATCTGGTCGCTCGTCTGC	CGAACGTCGCCACCTTGTTC	60.0
OsPIN2	CAACACCTACTCCAGCCTC	TGGACCAGTCAAGAACCTC	55.0
OsPIN9	GATACAAGATAGCGTCGTTCTC	ATGATGTCTGCGTGGACCT	55.5
