# Supplementary material for: Association Analysis of Tiller-Related Traits with EST-SSR Markers in Psathyrostachys juncea
Source: Genes (Basel). 2023 Oct 21;14(10):1970. doi: 10.3390/genes14101970 (PMC10606050; doi:10.3390/genes14101970)
Supplement: Supplementary file 1 [file genes-14-01970-s001.zip › Table S2.pdf]

Table S2 Genetic diversity analysis using SSR markers of *P. juncea*

Primer	Na	Ne	*I*	H_0	*He*	PIC	Primer	Na	Ne	*I*	H_0	*He*	PIC
81	2.000	1.997	0.692	0.876	0.499	0.375	94279	2.000	1.994	0.692	0.480	0.499	0.374
516	1.500	1.117	0.169	0.103	0.095	0.272	99009	2.000	1.475	0.503	0.317	0.322	0.372
2249	2.000	2.000	0.693	0.455	0.500	0.375	114416	2.000	1.548	0.539	0.420	0.354	0.291
002947A	2.000	1.994	0.692	0.586	0.499	0.374	120572	2.000	1.687	0.588	0.170	0.401	0.318
4435	2.000	1.532	0.531	0.291	0.347	0.324	126480	2.000	1.996	0.692	0.067	0.499	0.374
6022	2.000	1.425	0.474	0.325	0.298	0.253	127259	2.000	1.954	0.681	0.735	0.488	0.369
006181A	2.000	1.871	0.658	0.228	0.466	0.362	128934	2.000	1.652	0.578	0.463	0.390	0.313
8649	2.000	1.792	0.633	0.404	0.441	0.343	132067	2.000	1.395	0.441	0.326	0.274	0.233
13109	2.000	1.721	0.606	0.255	0.416	0.326	132162	1.500	1.458	0.335	0.210	0.239	0.182
13558	1.333	1.030	0.059	0.028	0.027	0.026	133160	2.000	2.000	0.693	0.800	0.400	0.375
016533C	1.500	1.177	0.215	0.122	0.131	0.114	134436	2.000	1.901	0.666	0.336	0.473	0.361
18679	2.000	1.904	0.667	0.530	0.474	0.361	135127	2.000	1.734	0.614	0.577	0.423	0.366
019368C	2.000	1.818	0.638	0.603	0.446	0.346	137131	2.000	1.592	0.496	0.208	0.330	0.262
022073A	2.000	1.991	0.691	0.681	0.498	0.373	138171	2.000	1.480	0.502	0.248	0.322	0.270
22271	2.000	1.527	0.527	0.430	0.344	0.284	139768A	2.000	1.250	0.350	0.220	0.199	0.179
024537A	2.000	1.442	0.442	0.211	0.281	0.232	139898	2.000	1.676	0.593	0.545	0.403	0.322
25029	2.000	1.494	0.457	0.403	0.297	0.237	139974	1.500	1.328	0.293	0.272	0.198	0.159
26249	2.000	2.000	0.693	0.426	0.500	0.375	140425	2.000	1.591	0.546	0.258	0.363	0.181
26620	2.000	1.732	0.592	0.293	0.407	0.319	141012	2.000	1.133	0.249	0.140	0.130	0.121
27806	2.000	1.886	0.663	0.625	0.470	0.360	141141	2.000	1.178	0.284	0.165	0.151	0.140
028161A	2.000	1.999	0.693	0.419	0.500	0.375	142295B	2.000	1.263	0.256	0.221	0.178	0.160
28324	2.000	1.336	0.383	0.104	0.233	0.201	142346	2.000	1.454	0.491	0.135	0.312	0.263
28410	2.000	1.999	0.693	0.113	0.500	0.375	142484	2.000	1.582	0.542	0.314	0.359	0.292
28553	2.000	1.725	0.611	0.578	0.420	0.334	142585	2.000	1.602	0.563	0.211	0.376	0.305
029223A	2.000	1.984	0.689	0.766	0.496	0.373	142612A	2.000	1.558	0.544	0.392	0.358	0.294
029223B	2.000	1.974	0.687	0.529	0.493	0.372	143409	2.000	1.688	0.598	0.301	0.408	0.324
29697	2.000	1.639	0.536	0.392	0.360	0.286	144127	2.000	1.149	0.253	0.140	0.130	0.121
030766B	2.000	1.173	0.279	0.136	0.148	0.137	144827	2.000	1.773	0.627	0.366	0.435	0.340
35661	2.000	1.227	0.328	0.081	0.183	0.166	144926B	2.000	1.803	0.631	0.286	0.440	0.342
37119	2.000	1.836	0.644	0.459	0.452	0.349	146991	2.000	1.769	0.623	0.537	0.432	0.338
37353	1.500	1.500	0.347	0.125	0.250	0.188	149977	2.000	1.923	0.672	0.361	0.479	0.364
38976	2.000	1.366	0.314	0.303	0.213	0.168	163142	2.000	1.819	0.642	0.278	0.450	0.349
40957	2.000	1.866	0.657	0.449	0.464	0.356	163639	2.000	1.711	0.594	0.596	0.406	0.321
43242	2.000	1.772	0.627	0.642	0.436	0.341	165982	2.000	1.771	0.627	0.577	0.435	0.341
043637B	1.500	1.499	0.346	0.244	0.250	0.187	167041	2.000	1.733	0.608	0.191	0.419	0.330
44262	2.000	1.787	0.626	0.631	0.435	0.339	185875	2.000	1.909	0.669	0.599	0.476	0.363
45589	2.000	1.688	0.582	0.450	0.396	0.314	193064B	2.000	1.924	0.673	0.330	0.480	0.365
045600A	2.000	1.263	0.363	0.132	0.208	0.187	194430	1.500	1.349	0.301	0.094	0.206	0.163
046829B	2.000	1.984	0.689	0.204	0.496	0.373	194938	2.000	1.550	0.444	0.312	0.296	0.232
51628	2.000	1.570	0.499	0.331	0.330	0.264	*OsIPT4*	2.000	1.579	0.518	0.257	0.343	0.276
058749A	2.000	1.825	0.642	0.517	0.450	0.348	*OsIPT5*	2.000	1.585	0.556	0.214	0.369	0.301

Primer	Na	Ne	*I*	*H0*	*He*	PIC	Primer	Na	Ne	*I*	*H0*	*He*	PIC
60437	2.000	1.197	0.304	0.181	0.165	0.151	*OsIPT7*	2.000	1.987	0.690	0.851	0.497	0.373
61356	2.000	1.670	0.590	0.235	0.401	0.320	*OsIPT8*	2.000	1.596	0.561	0.200	0.373	0.304
63559	1.500	1.500	0.346	0.254	0.250	0.187	*OsCKX1*	2.000	1.738	0.616	0.463	0.425	0.335
64748	2.000	1.520	0.526	0.324	0.342	0.284	*OsCKX4*	2.000	1.832	0.647	0.431	0.454	0.351
65010	2.000	1.942	0.678	0.254	0.485	0.367	*OsCKX5*	2.000	1.468	0.490	0.347	0.313	0.262
65544	2.000	1.457	0.493	0.144	0.314	0.264	*OsCKX9*	2.000	1.806	0.630	0.548	0.440	0.341
66628	2.000	1.784	0.621	0.252	0.431	0.336	*OsYUCCA4*	2.000	1.191	0.298	0.176	0.160	0.148
66842	2.000	1.971	0.686	0.291	0.493	0.371	*OsYUCCA6*	2.000	1.786	0.622	0.203	0.433	0.337
67939	2.000	1.794	0.625	0.213	0.435	0.338	*OsRR6*	2.000	1.990	0.691	0.716	0.497	0.374
68444	1.500	1.496	0.346	0.339	0.249	0.187	*OsRR7*	2.000	1.628	0.574	0.014	0.386	0.311
69862	2.000	1.969	0.685	0.499	0.492	0.371	*OsRR11*	2.000	1.945	0.679	0.750	0.486	0.368
70047	1.667	1.663	0.461	0.270	0.332	0.249	*OsTB1*	2.000	1.996	0.692	0.246	0.499	0.374
70343	2.000	1.789	0.633	0.353	0.441	0.344	*D3*	2.000	1.808	0.631	0.626	0.441	0.342
71974	2.000	1.607	0.507	0.382	0.338	0.268	*D14*	2.000	1.696	0.598	0.363	0.408	0.324
72525	2.000	1.833	0.644	0.699	0.452	0.349	*OsD3*	2.000	1.647	0.581	0.397	0.392	0.315
072655A	2.000	1.660	0.560	0.179	0.379	0.301	*OsD10*	2.000	1.815	0.636	0.166	0.445	0.345
73069	2.000	1.980	0.688	0.265	0.495	0.372	*OsD27*	2.000	2.000	0.693	0.800	0.400	0.375
73311	2.000	1.970	0.686	0.560	0.492	0.371	*OsD17*	2.000	1.948	0.679	0.355	0.486	0.368
073848A	2.000	1.937	0.677	0.303	0.484	0.367	*OsMADS57*	2.000	1.629	0.574	0.336	0.386	0.311
074428A	2.000	1.673	0.583	0.445	0.395	0.315	*OsPIN1a*	2.000	1.708	0.605	0.333	0.414	0.329
74564	2.000	1.918	0.671	0.239	0.478	0.364	*OsPIN2*	2.000	1.982	0.688	0.076	0.495	0.373
075415B	2.000	1.982	0.689	0.301	0.496	0.373	*OsPIN9*	2.000	1.924	0.672	0.263	0.479	0.364
075587A	2.000	1.202	0.309	0.045	0.168	0.154	Mean	1.957	1.682	0.554	0.353	0.379	0.300

Na, Observed number of alleles; Ne, Effective number of alleles; *I*, Shannon's information index; *Ho*, observed heterozygosity; *He,* expected heterozygosity; PIC, Polymorphic information content
