# Supplementary material for: Association Analysis of Tiller-Related Traits with EST-SSR Markers in Psathyrostachys juncea
Source: Genes (Basel). 2023 Oct 21;14(10):1970. doi: 10.3390/genes14101970 (PMC10606050; doi:10.3390/genes14101970)
Supplement: Supplementary file 1 [file genes-14-01970-s001.zip › Table S3.pdf]

Table S3 Q value of 480 *P. juncea* individuals in five groups

No.	Q1	Q2	Q3	Q4	Q5	Group	No.	Q1	Q2	Q3	Q4	Q5	Group
1	0.002	0.002	0.004	0.990	0.003	4	241	0.001	0.001	0.038	0.001	0.958	5
2	0.001	0.001	0.001	0.996	0.001	4	242	0.001	0.001	0.006	0.001	0.992	5
3	0.001	0.001	0.001	0.996	0.001	4	243	0.001	0.001	0.005	0.001	0.993	5
4	0.001	0.001	0.001	0.997	0.001	4	244	0.001	0.001	0.005	0.001	0.993	5
5	0.001	0.001	0.001	0.995	0.001	4	245	0.002	0.001	0.979	0.001	0.017	3
6	0.001	0.001	0.001	0.995	0.001	4	246	0.001	0.001	0.994	0.001	0.004	3
7	0.002	0.001	0.001	0.995	0.001	4	247	0.002	0.001	0.978	0.001	0.017	3
8	0.001	0.001	0.002	0.993	0.003	4	248	0.001	0.001	0.992	0.001	0.006	3
9	0.001	0.001	0.001	0.997	0.001	4	249	0.003	0.001	0.986	0.006	0.004	3
10	0.002	0.001	0.001	0.995	0.001	4	250	0.004	0.002	0.953	0.001	0.041	3
11	0.001	0.002	0.001	0.995	0.001	4	251	0.001	0.001	0.988	0.001	0.009	3
12	0.002	0.001	0.001	0.995	0.001	4	252	0.001	0.001	0.995	0.000	0.003	3
13	0.001	0.002	0.001	0.996	0.001	4	253	0.003	0.002	0.979	0.001	0.015	3
14	0.003	0.001	0.002	0.992	0.002	4	254	0.001	0.001	0.993	0.000	0.005	3
15	0.002	0.001	0.003	0.985	0.008	4	255	0.002	0.001	0.943	0.002	0.053	3
16	0.001	0.001	0.002	0.994	0.002	4	256	0.001	0.001	0.982	0.001	0.015	3
17	0.002	0.002	0.002	0.992	0.002	4	257	0.003	0.001	0.447	0.001	0.548	5
18	0.001	0.001	0.001	0.994	0.002	4	258	0.001	0.001	0.994	0.001	0.004	3
19	0.002	0.001	0.001	0.995	0.001	4	259	0.001	0.001	0.997	0.000	0.001	3
20	0.003	0.002	0.001	0.993	0.001	4	260	0.001	0.001	0.994	0.000	0.004	3
21	0.001	0.001	0.001	0.996	0.001	4	261	0.001	0.001	0.996	0.000	0.003	3
22	0.002	0.002	0.001	0.994	0.001	4	262	0.001	0.001	0.995	0.000	0.003	3
23	0.002	0.001	0.001	0.996	0.001	4	263	0.001	0.001	0.994	0.000	0.004	3
24	0.002	0.002	0.001	0.995	0.001	4	264	0.001	0.001	0.990	0.001	0.008	3
25	0.001	0.001	0.001	0.996	0.002	4	265	0.001	0.001	0.996	0.001	0.002	3
26	0.001	0.005	0.002	0.990	0.003	4	266	0.001	0.001	0.992	0.000	0.006	3
27	0.001	0.001	0.001	0.996	0.001	4	267	0.001	0.001	0.995	0.000	0.003	3
28	0.001	0.001	0.001	0.995	0.001	4	268	0.001	0.001	0.993	0.000	0.005	3
29	0.002	0.001	0.001	0.996	0.001	4	269	0.001	0.000	0.996	0.000	0.002	3
30	0.001	0.001	0.002	0.994	0.002	4	270	0.001	0.001	0.993	0.001	0.005	3
31	0.001	0.001	0.008	0.983	0.007	4	271	0.001	0.001	0.995	0.000	0.003	3
32	0.001	0.001	0.002	0.994	0.002	4	272	0.001	0.001	0.995	0.000	0.004	3
33	0.001	0.001	0.002	0.995	0.001	4	273	0.001	0.001	0.996	0.000	0.002	3
34	0.001	0.001	0.001	0.997	0.001	4	274	0.001	0.001	0.995	0.001	0.003	3
35	0.002	0.002	0.001	0.994	0.001	4	275	0.001	0.001	0.994	0.001	0.004	3
36	0.001	0.001	0.001	0.995	0.001	4	276	0.001	0.001	0.995	0.001	0.003	3
37	0.002	0.001	0.002	0.991	0.003	4	277	0.001	0.001	0.985	0.001	0.013	3

No.	Q1	Q2	Q3	Q4	Q5	Group	No.	Q1	Q2	Q3	Q4	Q5	Group
38	0.003	0.001	0.001	0.995	0.001	4	278	0.002	0.002	0.992	0.001	0.003	3
39	0.002	0.001	0.001	0.993	0.002	4	279	0.001	0.001	0.993	0.000	0.005	3
40	0.001	0.001	0.001	0.997	0.001	4	280	0.001	0.001	0.984	0.001	0.013	3
41	0.003	0.001	0.001	0.995	0.001	4	281	0.001	0.001	0.994	0.001	0.003	3
42	0.002	0.001	0.002	0.992	0.004	4	282	0.001	0.001	0.996	0.001	0.002	3
43	0.002	0.001	0.001	0.994	0.002	4	283	0.001	0.001	0.996	0.001	0.002	3
44	0.003	0.001	0.000	0.995	0.001	4	284	0.001	0.001	0.994	0.001	0.005	3
45	0.002	0.001	0.001	0.995	0.001	4	285	0.001	0.000	0.997	0.000	0.001	3
46	0.001	0.001	0.000	0.996	0.001	4	286	0.001	0.001	0.996	0.000	0.002	3
47	0.001	0.001	0.001	0.996	0.001	4	287	0.001	0.001	0.995	0.001	0.002	3
48	0.002	0.001	0.001	0.996	0.001	4	288	0.001	0.001	0.994	0.000	0.004	3
49	0.002	0.001	0.001	0.995	0.001	4	289	0.005	0.960	0.005	0.020	0.009	2
50	0.005	0.001	0.001	0.992	0.001	4	290	0.002	0.984	0.005	0.004	0.004	2
51	0.004	0.001	0.001	0.993	0.001	4	291	0.003	0.988	0.005	0.002	0.002	2
52	0.004	0.002	0.002	0.991	0.002	4	292	0.003	0.991	0.001	0.004	0.001	2
53	0.002	0.001	0.004	0.992	0.002	4	293	0.003	0.992	0.002	0.002	0.001	2
54	0.001	0.002	0.002	0.994	0.002	4	294	0.002	0.992	0.002	0.003	0.001	2
55	0.001	0.001	0.001	0.996	0.001	4	295	0.003	0.989	0.002	0.004	0.002	2
56	0.001	0.001	0.001	0.996	0.001	4	296	0.006	0.987	0.003	0.002	0.002	2
57	0.002	0.001	0.001	0.995	0.001	4	297	0.003	0.981	0.002	0.012	0.002	2
58	0.001	0.002	0.002	0.994	0.001	4	298	0.013	0.940	0.019	0.020	0.009	2
59	0.002	0.001	0.002	0.993	0.002	4	299	0.002	0.992	0.001	0.003	0.001	2
60	0.002	0.002	0.003	0.992	0.001	4	300	0.002	0.991	0.003	0.002	0.002	2
61	0.002	0.002	0.001	0.993	0.002	4	301	0.002	0.993	0.002	0.002	0.001	2
62	0.004	0.003	0.005	0.984	0.004	4	302	0.002	0.992	0.003	0.002	0.002	2
63	0.002	0.001	0.001	0.996	0.001	4	303	0.002	0.991	0.003	0.002	0.003	2
64	0.002	0.002	0.004	0.990	0.003	4	304	0.001	0.995	0.001	0.002	0.001	2
65	0.002	0.001	0.002	0.993	0.002	4	305	0.002	0.986	0.003	0.003	0.006	2
66	0.002	0.001	0.002	0.992	0.002	4	306	0.002	0.989	0.003	0.005	0.002	2
67	0.001	0.001	0.001	0.996	0.001	4	307	0.008	0.937	0.020	0.008	0.027	2
68	0.001	0.001	0.002	0.995	0.001	4	308	0.003	0.989	0.003	0.002	0.002	2
69	0.003	0.004	0.003	0.989	0.001	4	309	0.004	0.986	0.003	0.004	0.002	2
70	0.001	0.002	0.001	0.996	0.001	4	310	0.004	0.988	0.003	0.002	0.003	2
71	0.001	0.001	0.001	0.996	0.001	4	311	0.004	0.990	0.002	0.001	0.003	2
72	0.002	0.002	0.006	0.987	0.003	4	312	0.037	0.930	0.013	0.003	0.017	2
73	0.002	0.002	0.001	0.995	0.001	4	313	0.003	0.991	0.002	0.001	0.002	2
74	0.002	0.001	0.002	0.993	0.001	4	314	0.002	0.981	0.008	0.001	0.008	2
75	0.002	0.001	0.001	0.995	0.001	4	315	0.003	0.986	0.007	0.001	0.003	2
76	0.003	0.003	0.016	0.973	0.005	4	316	0.002	0.985	0.005	0.002	0.005	2

No.	Q1	Q2	Q3	Q4	Q5	Group	No.	Q1	Q2	Q3	Q4	Q5	Group
77	0.005	0.001	0.002	0.991	0.001	4	317	0.003	0.987	0.004	0.003	0.004	2
78	0.002	0.001	0.001	0.995	0.001	4	318	0.002	0.983	0.003	0.007	0.004	2
79	0.001	0.002	0.002	0.994	0.001	4	319	0.003	0.983	0.005	0.004	0.005	2
80	0.003	0.002	0.003	0.991	0.002	4	320	0.003	0.979	0.005	0.005	0.008	2
81	0.002	0.001	0.001	0.995	0.001	4	321	0.006	0.973	0.015	0.002	0.004	2
82	0.001	0.001	0.001	0.996	0.001	4	322	0.002	0.991	0.004	0.001	0.002	2
83	0.002	0.001	0.001	0.996	0.001	4	323	0.002	0.991	0.003	0.002	0.002	2
84	0.005	0.002	0.003	0.987	0.003	4	324	0.002	0.993	0.001	0.003	0.001	2
85	0.002	0.001	0.001	0.995	0.001	4	325	0.002	0.993	0.003	0.001	0.002	2
86	0.001	0.001	0.001	0.996	0.001	4	326	0.004	0.982	0.005	0.006	0.004	2
87	0.001	0.001	0.001	0.996	0.001	4	327	0.002	0.994	0.001	0.001	0.001	2
88	0.002	0.001	0.001	0.996	0.001	4	328	0.003	0.988	0.003	0.004	0.002	2
89	0.002	0.002	0.002	0.993	0.002	4	329	0.002	0.995	0.001	0.001	0.001	2
90	0.001	0.001	0.002	0.994	0.002	4	330	0.001	0.993	0.002	0.001	0.003	2
91	0.005	0.001	0.001	0.992	0.001	4	331	0.003	0.966	0.004	0.003	0.023	2
92	0.002	0.002	0.002	0.992	0.003	4	332	0.001	0.993	0.002	0.002	0.002	2
93	0.003	0.001	0.001	0.993	0.001	4	333	0.003	0.961	0.004	0.005	0.028	2
94	0.001	0.002	0.001	0.995	0.001	4	334	0.001	0.995	0.001	0.001	0.001	2
95	0.002	0.002	0.002	0.993	0.001	4	335	0.002	0.989	0.003	0.002	0.004	2
96	0.002	0.002	0.001	0.993	0.003	4	336	0.003	0.985	0.007	0.001	0.005	2
97	0.994	0.001	0.001	0.001	0.003	1	337	0.002	0.992	0.003	0.001	0.002	2
98	0.995	0.002	0.001	0.001	0.001	1	338	0.002	0.994	0.002	0.001	0.001	2
99	0.996	0.001	0.001	0.001	0.001	1	339	0.002	0.982	0.005	0.002	0.009	2
100	0.997	0.001	0.001	0.001	0.001	1	340	0.004	0.988	0.003	0.002	0.003	2
101	0.995	0.001	0.001	0.002	0.001	1	341	0.002	0.991	0.002	0.002	0.002	2
102	0.989	0.002	0.003	0.002	0.004	1	342	0.003	0.977	0.009	0.003	0.008	2
103	0.996	0.001	0.001	0.001	0.001	1	343	0.001	0.970	0.012	0.006	0.011	2
104	0.996	0.001	0.001	0.001	0.001	1	344	0.002	0.989	0.004	0.002	0.004	2
105	0.993	0.001	0.002	0.001	0.003	1	345	0.001	0.991	0.002	0.002	0.003	2
106	0.997	0.001	0.001	0.001	0.001	1	346	0.002	0.989	0.002	0.002	0.005	2
107	0.997	0.001	0.000	0.001	0.001	1	347	0.002	0.990	0.003	0.002	0.003	2
108	0.997	0.001	0.001	0.001	0.001	1	348	0.003	0.989	0.002	0.002	0.005	2
109	0.996	0.001	0.001	0.001	0.001	1	349	0.003	0.981	0.005	0.003	0.008	2
110	0.997	0.001	0.001	0.001	0.001	1	350	0.006	0.932	0.027	0.012	0.024	2
111	0.993	0.002	0.001	0.001	0.003	1	351	0.002	0.993	0.001	0.002	0.001	2
112	0.998	0.001	0.001	0.001	0.000	1	352	0.002	0.994	0.001	0.002	0.001	2
113	0.996	0.002	0.001	0.001	0.001	1	353	0.002	0.994	0.001	0.002	0.001	2
114	0.997	0.001	0.001	0.001	0.001	1	354	0.017	0.959	0.018	0.003	0.003	2
115	0.997	0.001	0.001	0.001	0.001	1	355	0.002	0.938	0.009	0.036	0.015	2

No.	Q1	Q2	Q3	Q4	Q5	Group	No.	Q1	Q2	Q3	Q4	Q5	Group
116	0.997	0.001	0.001	0.001	0.001	1	356	0.005	0.984	0.002	0.007	0.002	2
117	0.997	0.001	0.001	0.001	0.001	1	357	0.003	0.989	0.002	0.003	0.003	2
118	0.997	0.001	0.001	0.001	0.001	1	358	0.008	0.945	0.023	0.002	0.021	2
119	0.996	0.001	0.001	0.001	0.001	1	359	0.002	0.978	0.009	0.003	0.008	2
120	0.997	0.001	0.001	0.001	0.001	1	360	0.005	0.985	0.002	0.003	0.005	2
121	0.992	0.004	0.001	0.001	0.001	1	361	0.002	0.994	0.002	0.001	0.001	2
122	0.995	0.001	0.001	0.001	0.001	1	362	0.004	0.954	0.008	0.009	0.025	2
123	0.995	0.002	0.001	0.001	0.001	1	363	0.006	0.982	0.005	0.002	0.005	2
124	0.994	0.001	0.002	0.002	0.001	1	364	0.003	0.970	0.018	0.002	0.006	2
125	0.995	0.002	0.001	0.001	0.001	1	365	0.008	0.986	0.002	0.002	0.001	2
126	0.996	0.001	0.001	0.002	0.001	1	366	0.003	0.978	0.002	0.014	0.004	2
127	0.996	0.001	0.001	0.001	0.001	1	367	0.002	0.989	0.001	0.006	0.001	2
128	0.996	0.001	0.001	0.001	0.001	1	368	0.002	0.990	0.002	0.005	0.002	2
129	0.996	0.001	0.001	0.001	0.001	1	369	0.002	0.986	0.005	0.002	0.004	2
130	0.997	0.001	0.001	0.001	0.001	1	370	0.004	0.913	0.046	0.002	0.035	2
131	0.997	0.001	0.001	0.001	0.001	1	371	0.002	0.994	0.001	0.002	0.001	2
132	0.996	0.001	0.001	0.001	0.001	1	372	0.002	0.986	0.002	0.004	0.005	2
133	0.995	0.002	0.001	0.001	0.002	1	373	0.002	0.993	0.002	0.002	0.002	2
134	0.973	0.003	0.009	0.007	0.008	1	374	0.007	0.980	0.002	0.009	0.002	2
135	0.997	0.001	0.001	0.001	0.001	1	375	0.002	0.994	0.001	0.002	0.001	2
136	0.996	0.001	0.001	0.001	0.001	1	376	0.002	0.991	0.003	0.001	0.002	2
137	0.996	0.001	0.001	0.001	0.001	1	377	0.003	0.993	0.002	0.001	0.001	2
138	0.997	0.001	0.001	0.001	0.001	1	378	0.006	0.987	0.002	0.003	0.002	2
139	0.996	0.001	0.001	0.001	0.001	1	379	0.006	0.983	0.004	0.004	0.003	2
140	0.997	0.001	0.001	0.001	0.001	1	380	0.003	0.991	0.002	0.001	0.002	2
141	0.996	0.002	0.001	0.001	0.001	1	381	0.004	0.988	0.002	0.003	0.002	2
142	0.994	0.002	0.001	0.001	0.001	1	382	0.003	0.994	0.001	0.001	0.001	2
143	0.997	0.001	0.001	0.001	0.001	1	383	0.004	0.991	0.002	0.002	0.002	2
144	0.996	0.001	0.001	0.001	0.001	1	384	0.003	0.991	0.001	0.003	0.002	2
145	0.990	0.001	0.001	0.002	0.005	1	385	0.004	0.991	0.002	0.001	0.002	2
146	0.995	0.001	0.001	0.001	0.001	1	386	0.002	0.990	0.004	0.001	0.003	2
147	0.997	0.001	0.001	0.001	0.001	1	387	0.002	0.993	0.002	0.001	0.002	2
148	0.997	0.001	0.001	0.001	0.001	1	388	0.002	0.990	0.002	0.003	0.003	2
149	0.990	0.002	0.004	0.001	0.004	1	389	0.005	0.987	0.003	0.002	0.003	2
150	0.994	0.001	0.002	0.001	0.001	1	390	0.003	0.990	0.003	0.001	0.002	2
151	0.997	0.001	0.001	0.001	0.001	1	391	0.003	0.994	0.001	0.001	0.001	2
152	0.996	0.001	0.001	0.001	0.001	1	392	0.002	0.993	0.002	0.001	0.002	2
153	0.996	0.001	0.001	0.001	0.001	1	393	0.004	0.983	0.002	0.002	0.009	2
154	0.995	0.001	0.002	0.001	0.001	1	394	0.003	0.993	0.001	0.001	0.001	2

No.	Q1	Q2	Q3	Q4	Q5	Group	No.	Q1	Q2	Q3	Q4	Q5	Group
155	0.996	0.001	0.001	0.001	0.001	1	395	0.001	0.991	0.004	0.002	0.002	2
156	0.996	0.001	0.001	0.001	0.001	1	396	0.001	0.991	0.003	0.002	0.004	2
157	0.997	0.001	0.001	0.001	0.001	1	397	0.002	0.993	0.002	0.001	0.002	2
158	0.993	0.002	0.002	0.001	0.002	1	398	0.002	0.992	0.002	0.001	0.003	2
159	0.996	0.001	0.001	0.001	0.001	1	399	0.003	0.992	0.002	0.001	0.002	2
160	0.995	0.001	0.002	0.001	0.001	1	400	0.003	0.991	0.002	0.001	0.002	2
161	0.997	0.001	0.001	0.001	0.001	1	401	0.008	0.971	0.011	0.001	0.009	2
162	0.995	0.001	0.001	0.002	0.001	1	402	0.003	0.986	0.004	0.002	0.005	2
163	0.996	0.001	0.001	0.001	0.001	1	403	0.002	0.993	0.002	0.003	0.001	2
164	0.997	0.001	0.001	0.001	0.001	1	404	0.002	0.987	0.004	0.004	0.004	2
165	0.998	0.001	0.001	0.001	0.001	1	405	0.003	0.992	0.002	0.002	0.002	2
166	0.996	0.001	0.001	0.001	0.001	1	406	0.002	0.994	0.001	0.001	0.001	2
167	0.996	0.001	0.001	0.001	0.001	1	407	0.002	0.992	0.001	0.003	0.001	2
168	0.996	0.001	0.001	0.001	0.001	1	408	0.002	0.995	0.001	0.002	0.001	2
169	0.994	0.002	0.001	0.001	0.001	1	409	0.003	0.991	0.002	0.001	0.002	2
170	0.996	0.001	0.001	0.001	0.001	1	410	0.008	0.981	0.007	0.001	0.003	2
171	0.995	0.001	0.002	0.001	0.001	1	411	0.002	0.995	0.001	0.001	0.001	2
172	0.990	0.001	0.004	0.001	0.004	1	412	0.003	0.993	0.001	0.001	0.001	2
173	0.997	0.001	0.001	0.001	0.001	1	413	0.002	0.995	0.001	0.001	0.001	2
174	0.995	0.001	0.002	0.001	0.001	1	414	0.002	0.991	0.001	0.005	0.001	2
175	0.997	0.001	0.001	0.001	0.001	1	415	0.008	0.986	0.001	0.004	0.002	2
176	0.996	0.001	0.002	0.001	0.001	1	416	0.002	0.994	0.001	0.001	0.001	2
177	0.995	0.002	0.001	0.001	0.001	1	417	0.003	0.993	0.001	0.002	0.001	2
178	0.997	0.001	0.001	0.001	0.001	1	418	0.003	0.994	0.001	0.001	0.001	2
179	0.996	0.001	0.001	0.001	0.001	1	419	0.004	0.993	0.002	0.001	0.001	2
180	0.997	0.001	0.001	0.001	0.001	1	420	0.003	0.994	0.001	0.002	0.001	2
181	0.994	0.002	0.001	0.002	0.001	1	421	0.004	0.969	0.001	0.023	0.003	2
182	0.996	0.001	0.001	0.001	0.001	1	422	0.002	0.994	0.001	0.001	0.002	2
183	0.996	0.001	0.001	0.001	0.001	1	423	0.002	0.994	0.001	0.001	0.001	2
184	0.997	0.001	0.001	0.001	0.001	1	424	0.005	0.991	0.001	0.001	0.001	2
185	0.995	0.001	0.002	0.001	0.001	1	425	0.002	0.995	0.001	0.002	0.001	2
186	0.997	0.001	0.001	0.001	0.001	1	426	0.004	0.987	0.003	0.002	0.004	2
187	0.996	0.001	0.001	0.001	0.001	1	427	0.005	0.991	0.001	0.002	0.001	2
188	0.996	0.001	0.002	0.001	0.001	1	428	0.005	0.988	0.001	0.005	0.001	2
189	0.995	0.002	0.001	0.001	0.001	1	429	0.003	0.994	0.001	0.001	0.001	2
190	0.997	0.001	0.001	0.001	0.001	1	430	0.005	0.991	0.001	0.002	0.001	2
191	0.997	0.001	0.001	0.001	0.001	1	431	0.004	0.989	0.002	0.002	0.003	2
192	0.996	0.001	0.001	0.001	0.001	1	432	0.002	0.996	0.001	0.001	0.001	2
193	0.002	0.001	0.003	0.001	0.993	5	433	0.003	0.994	0.001	0.001	0.001	2

No.	Q1	Q2	Q3	Q4	Q5	Group	No.	Q1	Q2	Q3	Q4	Q5	Group
194	0.001	0.001	0.004	0.001	0.993	5	434	0.003	0.992	0.002	0.002	0.002	2
195	0.001	0.001	0.004	0.001	0.993	5	435	0.004	0.993	0.001	0.001	0.001	2
196	0.001	0.001	0.004	0.000	0.994	5	436	0.009	0.983	0.004	0.002	0.001	2
197	0.001	0.001	0.023	0.001	0.974	5	437	0.001	0.996	0.001	0.001	0.001	2
198	0.001	0.001	0.003	0.005	0.989	5	438	0.001	0.994	0.001	0.002	0.001	2
199	0.001	0.001	0.003	0.000	0.995	5	439	0.003	0.992	0.002	0.001	0.002	2
200	0.001	0.001	0.004	0.001	0.994	5	440	0.003	0.992	0.002	0.002	0.002	2
201	0.001	0.001	0.004	0.001	0.994	5	441	0.003	0.988	0.005	0.002	0.002	2
202	0.001	0.001	0.007	0.000	0.991	5	442	0.002	0.994	0.001	0.001	0.001	2
203	0.001	0.001	0.002	0.001	0.996	5	443	0.002	0.995	0.001	0.001	0.001	2
204	0.001	0.001	0.003	0.001	0.994	5	444	0.004	0.992	0.001	0.002	0.002	2
205	0.001	0.001	0.003	0.001	0.995	5	445	0.004	0.988	0.003	0.002	0.003	2
206	0.001	0.001	0.006	0.001	0.991	5	446	0.001	0.993	0.002	0.002	0.002	2
207	0.001	0.001	0.013	0.019	0.966	5	447	0.005	0.991	0.002	0.001	0.001	2
208	0.001	0.001	0.002	0.001	0.995	5	448	0.007	0.980	0.008	0.003	0.002	2
209	0.002	0.001	0.005	0.001	0.991	5	449	0.003	0.993	0.002	0.001	0.001	2
210	0.001	0.001	0.003	0.001	0.994	5	450	0.003	0.992	0.002	0.001	0.001	2
211	0.001	0.001	0.002	0.001	0.995	5	451	0.002	0.995	0.001	0.001	0.001	2
212	0.001	0.001	0.006	0.001	0.992	5	452	0.003	0.993	0.001	0.001	0.001	2
213	0.001	0.000	0.001	0.001	0.997	5	453	0.004	0.992	0.002	0.002	0.001	2
214	0.001	0.001	0.004	0.001	0.994	5	454	0.003	0.993	0.001	0.002	0.001	2
215	0.001	0.001	0.003	0.001	0.995	5	455	0.003	0.994	0.001	0.001	0.001	2
216	0.001	0.001	0.002	0.000	0.996	5	456	0.002	0.994	0.001	0.002	0.001	2
217	0.002	0.001	0.020	0.001	0.977	5	457	0.005	0.991	0.001	0.002	0.001	2
218	0.001	0.001	0.002	0.002	0.995	5	458	0.003	0.990	0.002	0.003	0.002	2
219	0.001	0.001	0.004	0.001	0.993	5	459	0.003	0.993	0.001	0.001	0.001	2
220	0.001	0.000	0.002	0.001	0.996	5	460	0.002	0.994	0.001	0.001	0.001	2
221	0.001	0.002	0.018	0.001	0.978	5	461	0.004	0.988	0.002	0.004	0.002	2
222	0.001	0.001	0.003	0.000	0.996	5	462	0.001	0.995	0.001	0.001	0.002	2
223	0.001	0.001	0.002	0.000	0.996	5	463	0.002	0.989	0.002	0.001	0.005	2
224	0.002	0.001	0.013	0.001	0.984	5	464	0.002	0.993	0.001	0.003	0.001	2
225	0.001	0.001	0.008	0.001	0.990	5	465	0.002	0.992	0.003	0.001	0.002	2
226	0.001	0.001	0.002	0.000	0.996	5	466	0.005	0.905	0.003	0.085	0.002	2
227	0.002	0.001	0.054	0.001	0.942	5	467	0.001	0.991	0.002	0.002	0.003	2
228	0.001	0.001	0.009	0.001	0.989	5	468	0.004	0.984	0.003	0.002	0.007	2
229	0.002	0.001	0.002	0.001	0.995	5	469	0.004	0.981	0.004	0.008	0.004	2
230	0.001	0.001	0.003	0.001	0.995	5	470	0.002	0.987	0.003	0.003	0.004	2
231	0.001	0.001	0.003	0.000	0.994	5	471	0.003	0.982	0.002	0.010	0.003	2
232	0.001	0.001	0.005	0.001	0.992	5	472	0.002	0.994	0.002	0.001	0.001	2

No.	Q1	Q2	Q3	Q4	Q5	Group	No.	Q1	Q2	Q3	Q4	Q5	Group
233	0.001	0.001	0.043	0.001	0.955	5	473	0.001	0.995	0.001	0.001	0.001	2
234	0.001	0.001	0.005	0.001	0.992	5	474	0.004	0.989	0.001	0.004	0.002	2
235	0.001	0.001	0.010	0.001	0.988	5	475	0.003	0.979	0.003	0.007	0.007	2
236	0.001	0.001	0.005	0.001	0.993	5	476	0.002	0.992	0.002	0.001	0.003	2
237	0.001	0.001	0.004	0.001	0.995	5	477	0.001	0.995	0.001	0.001	0.001	2
238	0.001	0.001	0.005	0.001	0.992	5	478	0.006	0.932	0.009	0.049	0.003	2
239	0.001	0.001	0.003	0.001	0.995	5	479	0.002	0.995	0.001	0.001	0.001	2
240	0.001	0.001	0.005	0.001	0.993	5	480	0.002	0.993	0.001	0.002	0.001	2
